# An Analysis of Factors Associated with Chronic Musculoskeletal Pain, Pain Management Preferences, Coping Strategies, and Health-Related Quality of Life Among Older People: A Cross-Cultural Study

**DOI:** 10.2147/JPR.S525968

**Published:** 2025-09-23

**Authors:** Ugur Cavlak, Ivan Jurak, Ligia Rusu, Guzin Kaya Aytutuldu, Mirjana Telebuh, Oana Bianca Budeanca-Babolea, Eylül Pınar Kısa, Zelimir Bertic, Eva Nicoleta Ilie, Gordana Grozdek Covcic, Denisa Piele, Margareta Begić, Mihai Marian Dragomir

**Affiliations:** 1 Department of Physiotherapy and Rehabilitation, Biruni University, Istanbul, Turkey; 2 Department of Physiotherapy, University of Applied Health Sciences, Zagreb, Croatia; 3 Department of Sports Medicine and Physiotherapy, University of Craiova, Craiova, Romania; 4 Department of Physiotherapy, University of Primorska, Izola, Slovenia; 5 Department of Ergotherapy, Istanbul Medipol University, Istanbul, Turkey; 6 Department of Nursing, University of Rijeka, Rijeka, Croatia; 7 Department of Public Health, Institute of Public Health of Bjelovar-Bilogora County, Bjelovar, Croatia

**Keywords:** pain measurements, aged, coping behavior, quality of life, cross-cultural comparison

## Abstract

**Introduction:**

Chronic musculoskeletal pain is common among older adults (OAs). This study aimed to identify and describe the characteristics of chronic musculoskeletal pain, pain management, and coping strategies among OAs from Turkey, Croatia, and Romania. The study also aimed to explore cultural differences in pain experience and management.

**Methods:**

A total of 337 OAs with chronic pain participated: 100 from Turkey, 131 from Croatia and 106 from Romania. The mean age was 76.3 years in Croatia, 73.1 years in Romania, and 74.0 years in Turkey. The majority of participants in Croatia and Romania were female, while the majority of participants in Turkey were male. A structured questionnaire was used to explore four main topics, including factors influencing pain, coping strategies, management strategies, and health-related quality of life.

**Results:**

Significant differences in pain-related factors, pain management, and coping strategies were found between the three countries (p≤0.05). Physical activity, relaxation exercises, and warm showers/hot packs were associated with pain reduction. Climbing stairs was a significant pain increasing factor in both Romania and Turkey, with participants in these countries more than 2.5 times more likely to report it as a pain increasing factor compared to Croatia. Sitting had the opposite effect in Romania and Turkey. Significant predictors included higher visual analog scale-VAS scores, which were associated with worse self-rated health. Country of origin also influenced health perceptions, with Romanians less likely to report better health than Croatians.

**Discussion:**

The findings highlight the cultural implications of pain perception and management. They reveal that older adults’ coping strategies and health-related quality of life are shaped not only by physical factors but also by beliefs and health perceptions unique to each country and influenced by culture. Education, including physical activity, medication use, and non-pharmacological methods like physiotherapy and interventions that consider cultural context can enhance pain control and health among older adults.

## Introduction

The International Association on the Study of Pain (IASP) wrote the revised definition of pain as “An unpleasant sensory and emotional experience associated with, or resembling that associated with, actual or potential tissue damage”.^1^ The analysis of the Global Burden of Disease 2019 revealed that approximately 1.71 billion people globally are affected by musculoskeletal conditions.^2–4^ World Health Organization (WHO) reported that musculoskeletal health conditions pose a global threat to healthy ageing with considerable negative effects.^5–10^ The topic of this paper takes into consideration, also, a few aspects regarding the specific cultural faces related to psychosocial factors, access to health facilities, pain expression, and pain assessment tools. According to literature studies, we speak about the pain as a part of aging process; therefore, in some cultures, the older people consider that there is no need for medical intervention.^11^ In the same context exist cultural groups, which sustain that pain could be hidden in social life and also according to different social status, financial position is possible to develop depression.^12^ From a cultural and socioeconomic perspective, pain assessment tools vary across countries, reflecting differences in healthcare systems and cultural norms. Additionally, there are distinctions in how older adults engage in physical activity, access medical services to discuss chronic pain, and receive support from their families.^13^ We consider the approach to the cultural branch in chronic pain management could help us develop strategies for improving the quality of life of older people. Problem statement is focused on diversity of cultural, social, and economic levels that could influence the pain perception, pain experience, coping strategies, pain management, and quality of life in older adults (OAs). The research problem is how cultural, and societal factors influence pain management. The problem statement is the lack of culturally tailored approaches, underreporting pain due to cultural expectations and limited research on how cultural framing affects pain perception and coping mechanisms.

A survey^14^ revealed that most papers on pain in the elderly are related to original research on pain characteristics, with limited evidence regarding biological factors relevant to pain processing in the context of aging. Ageing has been shown to lead to changes in the sensory components of pain, particularly in women. In addition, there are controversial data on the influence of aging on pain mechanisms.^15–17^ Primarily, chronic diseases as comorbidities, but also other conditions as well as medication should be taken into account to estimate the relative contribution of chronic musculoskeletal pain (CMP) to disability, loss of functional independence and poor quality of life. CMP is a common medical and socioeconomic problem worldwide.^17^ The main factors shaping the interest in pain perception derive from demographic changes in developed countries, characterized by substantial increases in life expectancy, as well as high prevalence rates of CMP among OAs.^18^ The biopsychosocial model of chronic pain presents an integrated approach to CMP, taking into account differences in nociceptive processes and psychosocial factors.^19^

Grosman et al describe the bidirectional relationship between sarcopenia and CMP, and their results demonstrate that specific mechanisms such as cellular aging, chronic inflammation, and gender-related hormonal changes are associated with psychosocial factors such as depression and social isolation and create premises for increasing the level of pain perception and need a specific analysis of pain CMP behavior to design the intervention.^20^ According to some authors, we have to approach the CMP as multifactorial symptoms that requires analysis of numerous biological, psychological, and social factors that are involved in pain syndrome, because chronic pain is a diagnosis and requires effective and sustainable treatment strategies.^21^ Given the multitude of contributing factors closely linked to geriatric-specific syndromes, persistent pain in OAs is considered by some experts as a geriatric syndrome.^10^ The Geriatric Pain Measure has been developed to provide a multidimensional assessment of pain that is easy to apply and understand. In order to achieve effective pain management (PM) among OAs, it would be desirable to use standardized and validated pain assessment tools specifically tailored to the elderly.^22^,^23^ Although pain in multiple areas is very common in OAs, it remains an underappreciated and understudied pain characteristic that may represent a distinct pain condition with potential functional implications for this population. CMP is a common medical and socioeconomic problem worldwide.^17^ The most prevalent forms of CMP are low back pain and neck pain. CMP imposes a considerable impact on individuals’ physical activity levels and their overall health-related quality of life (HRQoL). The analysis of the Global Burden of Disease 2019 revealed that approximately 1.71 billion people globally are affected by musculoskeletal conditions.^2–4^ The WHO reported that musculoskeletal health conditions pose a global threat to healthy ageing, having considerable negative effects.^5–10^ CMP among the elderly has a high prevalence^24^ and the well-established association with significantly lower HRQoL. Moreover, there has been a lack of detailed understanding of the extent to which each factor determines HRQoL in OAs.^6^,^25–30^ The main factors shaping the interest in pain perception derive from demographic changes in developed countries, characterized by substantial increases in life expectancy, as well as high prevalence rates of CMP among OAs.^18^ Unfortunately, pain may be undertreated or inadequately managed in OAs for a number of reasons, including lack of pain identification, false beliefs that pain is part of the aging process, cognitive impairments with pain variables, underreporting by patients, and limited time in clinical practice to address pain in the context of comorbidities.^31^,^32^

Holistic approach of CMP has to be done by practical application of the biopsychosocial model, which creates bridges between biomedical and social models and helps in analysis of the influences of biological, mental, and social factors on health. It is widely used in chronic pain management, mental health, and rehabilitation.^33^

This study aimed to identify the characteristics of CMP, PM, and coping strategies (CS) among OAs, while exploring cultural differences in pain experience, PM, and health perception. Based on our documentation, we consider that it is important to study how biopsychosocial integration influences pain perception, identifies specific factors in each country, and how it is possible to design strategies for personalized pain management, much more because pain perception and coping strategies depend on and vary across cultures and socioeconomic backgrounds.

A literature analysis can help to identify key factors to consider. For example, studies have highlighted that a comprehensive assessment incorporating biological, psychological and social interventions is required for chronic pain in older adults. At the same time, self-management strategies seem to be important because OAs who are active in their pain management and are interested in physical activity experience better outcomes.^34^ Research has also found that self-rated health is closely linked to pain levels, with individuals experiencing higher pain intensity often reporting poorer overall health.^35^

Practical experience and literature analysis demonstrate to us that there is a need to have a study regarding how cultural and societal differences can influence pain management strategies and pain perception. Also, cross-cultural pain expression and reporting are aspects that influence whether individuals openly express pain or suppress it.

At the same time, attitudes of older people against physical activity are different in each country, and there is a need to have a holistic approach to this aspect that will generate possible solutions for intervention. Our study also proposes to demonstrate the importance of an interdisciplinary approach to pain in older peoples.

The research question was as follows: How do the coping and pain management strategies that older adults use to manage musculoskeletal pain vary across cultural contexts, and what impact these strategies have on health-related quality of life (HRQoL)? This study aims to examine how older adults perceive musculoskeletal pain within specific cultural and societal contexts. It seeks to identify the pain management strategies they use and explore how cultural factors influence pain modulation. These factors include those that exacerbate or alleviate pain in ageing, coping mechanisms, pain management approaches, and health-related quality of life (HRQoL).

## Materials and Methods

The study included female (n=192) and male (n=145) OAs (n=337) aged between 65 and 85 years living in a nursing home, senior centers, or in her/his own residence. Study participants suffering from musculoskeletal pain on one or more body localizations were recruited from community-based organizations, mailings, social media, and from the physiotherapists’ database working in this field. Third-year and final-year physiotherapy students, who were informed about the aim of the study and how to collect data, filled out questionnaires for this study. The study was a cross-cultural study conducted simultaneously in Turkey (n=100), Romania (n=106), and Croatia (n=131). Using purposive, convenience sampling, the members of the research team from three countries worked together for this cross-sectional research. The study included OAs with chronic pain for at least 6 months, independent in activities of daily living, and independent in walking (with or without assistive walking devices). Elderly adults with communication problems were excluded from the study. All the participants fully understood the study and signed an informed consent form. The semi-structured interviews were conducted directly, face-to-face, with an average duration of approximately 15 min.

### Ethical Approval and Informed Consent

The present study received ethical approval and was conducted in accordance with the Declaration of Helsinki. Each country has received its own ethical approval from its institution. Each country has received its own ethical approval from its institutions (The Biruni University Non-Interventional Research Ethics Committee, Turkey: Number: 2023–81-13/Date: June 14, 2023; The Ethics Committee of Faculty of Physical Education and Sports, University of Craiova, Craiova, Romania: Number: 322/Date: August 2, 2023; The Ethics Committee University of Applied Health Sciences, Croatia: Number: 602–03/23-18/540, UB: 251-379-10-23-02/Date: August 30, 2023). Written informed consent was obtained from the participants.

To avoid bias due to seasonal effects, the study began at the same time in each country (early September to late December 2023). Data collection was therefore completed within a four-month period.

### Assessment Procedure

During each visit to a community, nursing home, or senior center, the researchers established contact with OAs who expressed interest in participating in the study. A structured questionnaire-based assessment was used in this study created by the authors of this study. The questionnaire was prepared in English and translated by the researchers in each country into their own language. Using structured and in-depth interviews, the study was conducted by physiotherapists, who have experience in qualitative methods. Four main themes were identified: (i) pain intensity measured using Visual Analogue Scale-VAS and the factors affecting pain such as increasing and decreasing factors in ageing; (ii) the coping strategies with pain in ageing; (iii) the management strategies in ageing; (iv) the health-related quality of life-related pain in ageing.^36–40^

This quantitative study employed a structured questionnaire-based assessment developed by the researchers based on relevant literature, administered to OAs who met the inclusion criteria (see Appendix 1). Demographics including age (year), gender (male/female), height (cm), weight (kg), body mass index-BMI score (kg/m^2^), marital status (married, single, never married, living with partner, living alone, widow), education level (illiterate, literate, primary school, high school, college, university level, postgraduate education), profession (white collar, blue collar, retirement, housewife, retirement-still working, other), exercise or sports habit (once a day, once a week, twice a week, three a week, more than three a week, everyday), living area (his/her residence, senior home, nursing home, other) were recorded on Section A of the survey.^4^,^36^,^39–41^

Section B consists of the pain assessment section, which evaluates pain localization, pain intensity, type of pain, factors affecting pain, coping with pain and management strategies.

To assess pain and its characteristics, pain reason, pain localization, pain intensity, pain duration during the day, pain frequency during the day, length of pain history, factors decreasing pain, factors increasing pain, coping strategies with pain, treatment for PM, medical coping strategies, taking pills daily were questioned and recorded.

The VAS is a tool for measuring subjective experiences like pain, discomfort, or other sensations, commonly used with OAs in clinical settings. It helps assess pain intensity, especially in those with communication challenges due to cognitive decline or sensory impairment. VAS is useful for evaluating pain severity in conditions like osteoarthritis, neuropathy, post-surgical pain, and cancer-related pain.^28^,^37^,^42–45^ In this study, participants rated their pain on an 11-point scale, from 0 (no pain) to 10 (worst pain imaginable). Scores were categorized as severe pain (7–10), moderate pain (4–6), mild pain (1–3), or no pain (0).^46^

**Figure 1:**
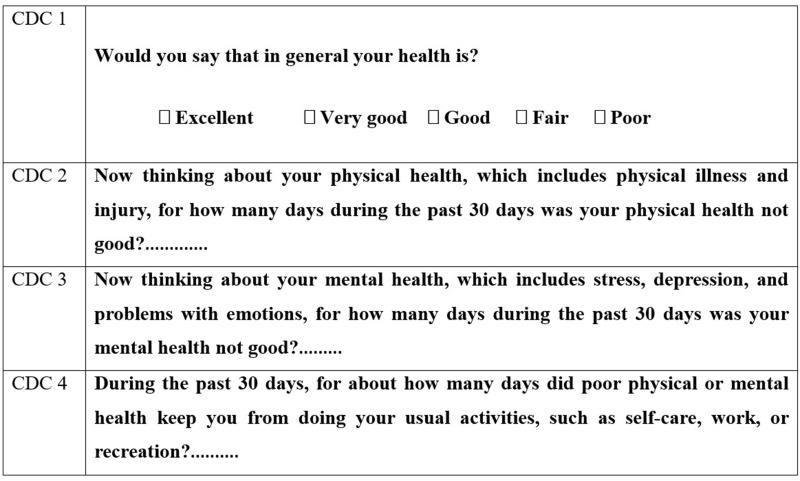
The CDC HRQOL-4, which is a brief set of survey-based questions designed to assess HRQOL – defined as “perceived physical and mental health over time”.

Section C included the Centers for Disease Control and Prevention Health-Related Quality of Life-4 (CDC HRQoL-4), which was developed to assess a person’s sense of well-being through four questions. A core set of these measures (the CDC HRQoL-4) asks about self-rated general health and the number of recent days when a person was physically unhealthy, mentally unhealthy, or limited in usual activities. A summary measure combines physically and mentally unhealthy days. It is a 4-question questionnaire that is easy to use in the OAs and assesses the quality of life. The brief standard CDC HRQoL-4 is now often used in surveys, surveillance systems, prevention research, and population health report cards. It includes a core set of four questions (Figure 1).^37^,^47^,^48^

### Statistical Analysis

Descriptive statistics were used to summarize the socio-demographic characteristics and pain-related factors across countries. To test differences in socio-demographic characteristics we employed Chi-square tests for categorical variables, such as gender and marital status, and Fisher’s exact test for variables with small, expected cell counts, such as education and living area. After testing the assumptions of distribution normality and homogeneity of variances, we used Welch’s ANOVA to compare continuous variables (age, height, weight, BMI) across countries due to unequal variances, followed by Games–Howell post-hoc tests to identify specific group differences. Logistic regression models were conducted to examine the associations in pain-decreasing/pain-increasing factors, pain self-coping strategies, and pain self-management behaviors, while controlling for age and BMI with “country” of participants being the main predictor of interest in these models. McFadden’s pseudo-R² values were reported to assess model fit, with odds ratios (OR) and accompanying 95% confidence intervals (CI). Reference category for predictor “country” was Croatia due to having the largest number of participants.

To analyze self-rated health status (CDC 1), measured on a five-level ordinal scale (“Excellent” to “Poor”), we used an ordinal logistic regression model (cumulative link model), with age, BMI, pain intensity (VAS), and country as predictors. The proportional odds assumption was tested, and though it held for continuous variables, it was violated for the categorical predictor of interest (Country). The model was retained due to its interpretability and stability, and this limitation is acknowledged. For the remaining CDC HRQoL-4, CDCs (CDC 2–4), which represent count-type responses (0–30 days) and violated normality assumptions (Shapiro–Wilk test), we applied the nonparametric Kruskal–Wallis test to assess differences across countries. Significant effects were followed by Dwass-Steel-Critchlow-Fligner post hoc tests for pairwise comparisons.

A post hoc power analysis was conducted via Monte Carlo simulation (1000 iterations, α = 0.05) to evaluate the ability of the logistic regression model to predict the use of painkillers to detect country-level effects, controlling for age and BMI (intercept = 4.273; β(age) = –0.042; β(BMI) = –0.069; sample sizes: Croatia n = 131, Romania n = 106, Turkey n = 100). This model was selected for power analysis due to its robust explanatory performance, as indicated by a McFadden’s R² of 0.383 and a highly significant omnibus test (χ² = 160, *p* < 0.001), suggesting excellent fit and meaningful variance explained. Simulated age and BMI values were drawn from normal distributions representing population characteristics. Power was estimated under varying hypothetical odds ratios (OR) for Romania and Turkey versus Croatia. The highest observed power at OR = 1.5 was 30.2% (Turkey); at OR = 2.0 it was 74.9% (Turkey); and at OR = 2.5, power reached 94.1% (Romania). These results suggest that while the study had limited power to detect small effects (OR = 1.5), it was sufficiently powered to detect moderate (OR = 2.0) and strong (OR = 2.5) country-level differences in pain self-management behavior.

The Type I error rate (α) was set at 5% (p < 0.05) to determine the statistical significance. Statistical analyses were conducted using *jamovi* (ver. 2.3.28)^49^ and R (ver. 4.3.1),^50^ depending on the analysis type. Data wrangling and post hoc power analysis were performed in R. The following analyses were conducted in *jamovi*: all tests of differences in sociodemographic characteristics, Welch’s ANOVA and associated post hoc comparisons, logistic regression models, and Kruskal–Wallis tests with Dwass-Steel-Critchlow-Fligner post hoc tests. The ordinal logistic regression model used to analyze self-rated health status (CDC 1), along with associated assumption testing, was performed in R using the *ordinal* package.^51^

To analyse self-rated health status (CDC 1), we used ordinal logistic regression model, with CDC 1 levels ordered as “excellent”, “very good”, “good”, “fair”, and “poor”. This model was also controlled for age and BMI, while Visual Analog Scale (VAS) for pain, and “country” were used as predictors. Model fit was assessed using a chi-square test for the overall model.

For the remaining CDC Health-Related Quality of Life measures (CDC 2, CDC 3, CDC 4), a Kruskal–Wallis test was used to examine differences across countries, as these variables did not meet normality assumptions, tested via Shapiro–Wilk *W*-test. Following significant Kruskal–Wallis tests, Dwass-Steel-Critchlow-Fligner post hoc tests were performed for pairwise country comparisons.

The Type I error rate (α) was set at 5% (p < 0.05) to determine statistical significance. Statistical analysis was performed using *jamovi* statistical software (ver. 2.3.28.0) and R statistical software (ver. 4.3.1).

## Results

### Descriptive results: Basic Sample Parameters, Pain Decreasing & Increasing Factors

A one-way ANOVA (Welch’s) analysis revealed significant differences in mean age (F (2, 219) = 7.02, p = 0.001), mean height (F (2, 214) = 9.39, p < 0.001), mean weight (F (2, 216) = 4.55, p = 0.012), and mean BMI (F (2, 212) = 17.32, p < 0.001) among participants from Croatia, Romania, and Turkey. Post-hoc comparisons using the GamesGames–Howell test indicated that Croatian participants were significantly older than both Romanian (mean difference = 3.19, p < 0.01) and Turkish participants (mean difference = 2.33, p = 0.029). Regarding BMI, Turkish participants had significantly higher BMI compared to both Croatian (mean difference = −3.10, p < 0.001) and Romanian participants (mean difference = −3.95, p < 0.001).

Marital status varied, with Turkey having the highest proportion of married individuals (71.0%), and Croatia and Romania having a majority of widowed or divorced individuals. Educational levels were lowest in Turkey, where 15% were illiterate and only 10% had a college degree. Most participants in Croatia and Romania were white-collar retirees, while over half of those from Turkey were housewives. Living arrangements also differed significantly, with 85% of participants in Turkey living in their own residence, compared to 51.1% in Croatia and 28.3% in Romania (Table 1).

**Table 1:** Descriptive Sample Parameters and Statistical Testing by Country

	Croatia (N=131)	Romania (N=106)	Turkey (N=100)	Test p-value
**Gender**				Χ^2^ <0.01
Male	31 (23.7%)	41 (38.7%)	73 (73.0%)	
Female	100 (76.3%)	65 (61.3%)	27 (27.0%)	
**Age (yrs)**				ANOVA <0.01
Mean (SD)	76.3 (7.20)	73.1 (6.23)	74.0 (6.51)	
Median [Min, Max]	76.0 [62.0, 92.0]	73.0 [65.0, 90.0]	72.0 [65.0, 85.0]	
**Height (m)**				ANOVA <0.01
Mean (SD)	1.65 (0.0929)	1.67 (0.0652)	1.62 (0.0927)	
Median [Min, Max]	1.63 [1.45, 1.90]	1.67 [1.50, 1.90]	1.60 [1.45, 1.88]	
**Weight (kg)**				ANOVA <0.01
Mean (SD)	74.1 (15.2)	72.9 (12.9)	78.8 (15.6)	
Median [Min, Max]	73.0 [39.0, 120]	71.0 [48.0, 120]	78.5 [50.0, 150]	
**Body Mass Index (BMI)**				ANOVA <0.01
Mean (SD)	27.1 (4.71)	26.2 (3.97)	30.2 (5.56)	
Median [Min, Max]	26.3 [16.0, 42.2]	26.0 [17.3, 39.2]	29.4 [19.5, 49.0]	
**Marital Status**				Fisher <0.01
Married	43 (32.8%)	29 (27.4%)	71 (71.0%)	
Single	5 (3.8%)	12 (11.3%)	29 (29.0%)	
Widow/divorced	79 (60.3%)	65 (61.3%)	0 (0%)	
**Education Level**				Fisher <0.01
Illiterate	0 (0%)	0 (0%)	15 (15.0%)	
Literate	2 (1.5%)	2 (1.9%)	15 (15.0%)	
Primary School	17 (13.0%)	37 (34.9%)	42 (42.0%)	
High School	73 (55.7%)	52 (49.1%)	18 (18.0%)	
College/ University	33 (25.2%)	12 (11.3%)	10 (10.0%)	
Postgraduate	6 (4.6%)	3 (2.8%)	0 (0%)	
**Profession**				Fisher <0.01
White collar, retired	76 (58.0%)	64 (60.4%)	17 (17.0%)	
Blue collar, retired	37 (28.2%)	36 (34.0%)	25 (25.0%)	
Housewife	8 (6.1%)	1 (0.9%)	54 (54.0%)	
Own business, retired	2 (1.5%)	0 (0%)	0 (0%)	
Still working	4 (3.1%)	4 (3.8%)	4 (4.0%)	
Other	4 (3.1%)	1 (0.9%)	0 (0%)	
**Living Area**				Fisher <0.01
Own residence	67 (51.1%)	30 (28.3%)	85 (85.0%)	
Nursing home	64 (48.9%)	76 (71.7%)	15 (15.0%)	

**Abbreviations**: Min, Minimum; Max, Maximum; SD, Standard Deviation; χ^2^, Chi-square test; ANOVA, Welch’s ANOVA test; Fisher, Fisher’s exact test.

Tables 2 and 3 show variables dealing with pain localization, pain duration, pain intensity, type of pain. The distribution of pain intensity measured by VAS (mean±SD) of the countries is as follows: Croatia (5.21±2.36), Romania (5.88±1.53), Turkey (5.46±1.81). These data show that older people living in all three countries reported moderate pain, according to the VAS.

**Table 2:** Pain Localization

Body Region	Yes (n)	Yes (%)	No (n)	No (%)
Neck	93	27.6%	244	72.4%
Shoulders	120	35.6%	217	64.4%
Upper back	76	22.6%	261	77.4%
Elbows	37	11.0%	300	89.0%
Wrists / Hands	56	16.6%	281	83.4%
Lower back	167	49.6%	170	50.4%
Hips / Thighs	93	27.6%	244	72.4%
Knees	170	50.4%	167	49.6%
Ankles / Feet	72	21.4%	265	78.6%

**Table 3:** Frequencies of Pain Characteristics

Category	Response	n	%
Pain Duration	Less than half hour	69	20.5%
	30 min – 60 min	85	25.2%
	More than 60 min	83	24.6%
	Whole day	76	22.6%
	Whole night	24	7.1%
Pain Frequency	Once in a day	82	24.3%
	Twice in a day	75	22.3%
	Three times in a day	68	20.2%
	More than three times in a day	112	33.2%
Pain History	Less than 6 months	24	7.1%
	6 months – 1 year	43	12.8%
	2 to 5 years	94	27.9%
	More than 5 years	176	52.2%

Table 4 shows the frequency distribution of factors influencing the decrease and increase in pain. Participants in all three countries reported that taking painkillers was the factor that most reduced pain, while standing for long periods was the factor that most increased pain. The frequency distribution of self-coping and self-management strategies for pain is shown in Table 5. Participants from all three countries preferred pharmacotherapy more in PM (Croatia: 73.3%; Romania: 58.5%; Turkey: 61.0%), while Long time in standing position was reported as the factor that increased pain the most.

**Table 4:** Frequency Distribution of Factors Influencing Pain Decrease and Increase

	Croatia (N=131)	Romania (N=106)	Turkey (N=100)
**Pain decreasing factors**			
**Sitting in a chair for a while**			
Yes	24 (18.3%)	21 (19.8%)	27 (27.0%)
No	107 (81.7%)	85 (80.2%)	73 (73.0%)
**Lying down in a bed or sofa**			
Yes	61 (46.6%)	67 (63.2%)	70 (70.0%)
No	70 (53.4%)	39 (36.8%)	30 (30.0%)
**Doing physical activity and exercise**			
Yes	52 (39.7%)	46 (43.4%)	7 (7.0%)
No	79 (60.3%)	60 (56.6%)	93 (93.0%)
**Doing relaxing exercises**			
Yes	36 (27.5%)	52 (49.1%)	11 (11.0%)
No	95 (72.5%)	54 (50.9%)	89 (89.0%)
**Taking pain killers**			
Yes	83 (63.4%)	78 (73.6%)	57 (57.0%)
No	48 (36.6%)	28 (26.4%)	43 (43.0%)
**Yoga, Pilates, dance, tai chi, swimming**			
Yes	6 (4.6%)	2 (1.9%)	0 (0%)
No	125 (95.4%)	104 (98.1%)	100 (100%)
**Warm shower, hot pack application**			
Yes	14 (10.7%)	17 (16.0%)	28 (28.0%)
No	117 (89.3%)	89 (84.0%)	72 (72.0%)
**Doing physical activity and exercise**			
Yes	17 (13.0%)	13 (12.3%)	16 (16.0%)
No	114 (87.0%)	93 (87.7%)	84 (84.0%)
**Pain increasing factors**			
**Long time in standing position**			
Yes	81 (61.8%)	73 (68.9%)	65 (65.0%)
No	50 (38.2%)	33 (31.1%)	35 (35.0%)
**Long time walking**			
Yes	50 (38.2%)	51 (48.1%)	62 (62.0%)
No	81 (61.8%)	55 (51.9%)	38 (38.0%)
**Long time lying in a bed**			
Yes	34 (26.0%)	14 (13.2%)	10 (10.0%)
No	97 (74.0%)	92 (86.8%)	90 (90.0%)
**Climbing the stairs**			
Yes	50 (38.2%)	63 (59.4%)	63 (63.0%)
No	81 (61.8%)	43 (40.6%)	37 (37.0%)
**Long time sitting**			
Yes	51 (38.9%)	60 (56.6%)	24 (24.0%)
No	80 (61.1%)	46 (43.4%)	76 (76.0%)
**Carrying heavy objects**			
Yes	56 (42.7%)	32 (30.2%)	47 (47.0%)
No	75 (57.3%)	74 (69.8%)	53 (53.0%)
**Pushing objects**			
Yes	20 (15.3%)	0 (0%)	25 (25.0%)
No	111 (84.7%)	106 (100%)	75 (75.0%)

**Table 5:** Frequency Distribution of Pain Self-Coping and Pain Self-Management Strategies

	Croatia (N=131)	Romania (N=106)	Turkey (N=100)
**Pain self-coping**			
**Doing exercise**			
Yes	52 (39.7%)	0 (0%)	1 (1.0%)
No	79 (60.3%)	106 (100%)	99 (99.0%)
**Taking a warm shower**			
Yes	17 (13.0%)	65 (61.3%)	11 (11.0%)
No	114 (87.0%)	41 (38.7%)	89 (89.0%)
**Taking a short walk**			
Yes	48 (36.6%)	21 (19.8%)	26 (26.0%)
No	83 (63.4%)	85 (80.2%)	74 (74.0%)
**Heat application**			
Yes	7 (5.3%)	43 (40.6%)	18 (18.0%)
No	124 (94.7%)	63 (59.4%)	82 (82.0%)
**Cold application**			
Yes	14 (10.7%)	22 (20.8%)	40 (40.0%)
No	117 (89.3%)	84 (79.2%)	60 (60.0%)
**Taking painkillers**			
Yes	90 (68.7%)	5 (4.7%)	9 (9.0%)
No	41 (31.3%)	101 (95.3%)	91 (91.0%)
**Pain self-Management**			
**Pharmacotherapy**			
Yes	96 (73.3%)	62 (58.5%)	61 (61.0%)
No	35 (26.7%)	44 (41.5%)	39 (39.0%)
**Massage therapy**			
Yes	28 (21.4%)	21 (19.8%)	31 (31.0%)
No	103 (78.6%)	85 (80.2%)	69 (69.0%)
**Physiotherapy**			
Yes	64 (48.9%)	88 (83.0%)	31 (31.0%)
No	67 (51.1%)	18 (17.0%)	69 (69.0%)
**Osteopathy / manual therapy**			
Yes	4 (3.1%)	22 (20.8%)	7 (7.0%)
No	127 (96.9%)	84 (79.2%)	93 (93.0%)
**Spa and balneotherapy**			
Yes	5 (3.8%)	29 (27.4%)	8 (8.0%)
No	126 (96.2%)	77 (72.6%)	92 (92.0%)
**Complementary therapies**			
Yes	1 (0.8%)	28 (26.4%)	4 (4.0%)
No	130 (99.2%)	78 (73.6%)	96 (96.0%)
**Acupuncture and dry needling**			
Yes	3 (2.3%)	8 (7.5%)	4 (4.0%)
No	128 (97.7%)	98 (92.5%)	96 (96.0%)

### Inferential Statistics: Logit Models for Pain Decreasing/Increasing Factors and Pain Self-Coping/Pain Self-Management

Table 6 presents logistic regression overall results for various pain-decreasing and pain-increasing factors, as well as pain-coping and pain-management strategies. Among the pain-decreasing factors, models for *doing physical activity/exercise* (R²McF = 0.117, p < 0.01), *doing relaxing exercises* (R²McF = 0.096, p < 0.01), and *warm shower/hot pack application* (R²McF = 0.046, p < 0.01) demonstrated statistically significant associations with pain reduction, albeit with modest explanatory power, as evidenced by McFadden’s pseudo-R². Similarly, *lying down in a bed or sofa* (R²McF = 0.045, p < 0.01) also showed a significant association, but with a relatively weak model fit. Among pain-increasing factors, *pushing an object* stood out with a higher explanatory power (R²McF = 0.154, p < 0.01), indicating a stronger association, while other factors *like long time sitting, climbing the stairs*, and *long-time lying* exhibited lower but still significant model fits (R²McF between 0.045 and 0.053). For pain self-coping strategies, significant and well-fitted models include *doing exercise* (R²McF = 0.38, p < 0.01), *taking a warm shower* (R²McF = 0.23, p < 0.01), and *taking painkillers* (R²McF = 0.383, p < 0.01), indicating strong associations. Other coping strategies like *heat application* and *cold application* had moderate model fits (R²McF around 0.117 to 0.150, p < 0.01), suggesting moderate associations. In terms of pain self-management, the models for *physiotherapy* (R²McF = 0.137, p < 0.01), *spa and balneotherapy* (R²McF = 0.141, p < 0.01), and *complementary therapy* (R²McF = 0.235, p < 0.01) demonstrated significant and moderately strong associations. On the other hand, models like *sitting in a chair* for a while, *doing yoga/Pilates*, and *massage therapy* were not statistically significant, with R²McF values indicating very low explanatory power. To summarize, the findings show a stronger and more significant impact of physical and complementary strategies on PM.

**Table 6:** Logistic Regression Models for Pain Decreasing/Increasing Factors and Pain Coping/Management Strategies (Overall Model Tests for y = Age + BMI + Country)

Model	*R²*McF	χ² Statistic	p - value
**Pain decreasing factors**			
Sitting in a chair for a while	0.01	4.46	0.35
Lying down in a bed or sofa	0.05	20.7	<0.01
Doing physical activity / exercise	0.12	49.0	<0.01
Doing relaxing exercises	0.10	39.2	<0.01
Taking pain killers	0.02	6.78	0.15
Doing yoga, Pilates, dance, tai chi, swimming	0.11	8.12	0.09
Warm shower, hot pack application	0.05	14.5	<0.01
**Pain decreasing factors**			
Doing physical activity and exercise	0.02	3.94	0.41
Long time in standing position	0.01	5.22	0.27
Long time walking	0.03	14.7	<0.01
Long time lying	0.05	16.4	<0.01
Climbing the stairs	0.05	20.8	<0.01
Long time sitting	0.05	23.9	<0.01
Carrying heavy objects	0.02	9.16	0.06
Pushing an object	0.15	40.7	<0.01
**Pain self-coping**			
Doing exercise	0.38	111	<0.01
Taking a warm shower	0.23	89.6	<0.01
Taking a short walk	0.04	16.5	<0.01
Heat application	0.15	50.7	<0.01
Cold application	0.12	42.1	<0.01
Taking painkillers	0.38	160	<0.01
**Pain self-management**			
Pharmacotherapy	0.05	19.6	<0.01
Massage therapy	0.01	4.82	0.31
Physiotherapy	0.14	63.9	<0.01
Osteopathy / manual therapy	0.11	24.1	<0.01
Spa and balneotherapy	0.14	35.7	<0.01
Complementary therapy	0.24	50.7	<0.01
Acupuncture and dry needling	0.04	4.84	0.30

**Abbreviations**: *R²*McF, McFadden’s pseudo-R².

Table 7 shows the logistic regression model coefficients for country differences in pain decreasing and increasing factors. Age and BMI, as they are used only as a controlling covariate, are not presented.

**Table 7:** Logistic Regression Model Coefficients of Country Predictor for Pain Decreasing & Increasing Factors

	Estimate	SE	Z	p	OR	LI	UI
**Pain decreasing factors**							
**Lying down in a bed or sofa**							
Romania – Croatia	0.70	0.28	2.54	<0.01	2.02	1.73	3.64
Turkey – Croatia	0.80	0.29	2.76	<0.01	2.23	1.26	3.93
**Doing physical activity and exercise**	** **	** **	** **	** **	** **	** **	** **
Romania – Croatia	0.23	0.28	0.82	0.41	1.25	0.73	2.15
Turkey – Croatia	−2.07	0.44	−4.71	<0.01	0.13	0.05	0.30
**Doing relaxing exercises**							
Romania – Croatia	0.95	0.29	3.32	<0.01	2.58	1.47	4.51
Turkey – Croatia	−1.23	0.39	−3.16	0.00	0.29	0.14	0.63
**Warm shower, hot pack application**							
Romania – Croatia	0.38	0.40	0.96	0.34	1.46	0.67	3.17
Turkey – Croatia	1.07	0.37	2.87	0.00	2.91	1.40	6.04
**Pain increasing factors**							
**Long time walking**							
Romania – Croatia	0.43	0.27	1.59	0.11	1.54	0.90	2.63
Turkey – Croatia	0.88	0.28	3.11	0.00	2.41	1.39	4.21
**Long time lying in a bed**							
Romania – Croatia	−0.70	0.36	−1.95	0.05	0.50	0.25	1.00
Turkey – Croatia	−1.02	0.40	−2.54	0.01	0.36	0.16	0.79
**Climbing the stairs**							
Romania – Croatia	0.96	0.28	3.47	<0.01	2.62	1.52	4.51
Turkey – Croatia	0.94	0.29	3.30	<0.01	2.57	1.47	4.49
**Long time sitting**							
Romania – Croatia	0.67	0.27	2.47	0.01	1.96	1.15	3.34
Turkey – Croatia	−0.72	0.31	−2.34	0.02	0.49	0.27	0.89
**Pushing an object**							
Romania – Croatia	–	–	–	–	–	–	–
Turkey – Croatia	0.65	0.35	1.83	0.07	1.91	0.96	3.83

**Abbreviations**: Estimate, log odds ratio; SE, standard error (log odds ratio); Z, Z value; p, p-value; OR, odd ratio; LI, lower interval of 95% confidence interval (odds ratio); UI, upper interval of 95% confidence interval (odds ratio).

For pain-decreasing factors, *lying down in a bed or sofa* was significantly associated with both Romania and Turkey compared to Croatia, with OR of 2.02 (p < 0.01) for Romania and 2.23 (p < 0.01) for Turkey, indicating that individuals in both countries were over twice as likely to report this as a pain-decreasing factor. *Doing physical activity and exercise* only showed significant negative association for Turkey compared to Croatia (OR = 0.13, p < 0.001), meaning that physical activity and exercise are less likely to be a perceived pain decreasing factor. *Doing relaxing exercises* also showed a large effect in Romania (OR = 2.58, p < 0.001) but a significant negative association in Turkey (OR = 0.29, p < 0.01), suggesting it is more frequently chosen in Romania and less in Turkey compared to Croatia. Additionally, *taking a warm shower* or *using a hot pack* had a strong association in Turkey (OR = 2.91, p < 0.01), indicating that it was more likely to be reported as a pain-reducing strategy in Turkey.

For pain-increasing factors, *climbing the stairs* was significant in both Romania and Turkey, with nearly identical ORs (2.62 for Romania and 2.57 for Turkey, both p < 0.001), meaning individuals in both countries were over 2.5 times more likely to report this as a pain-increasing factor compared to Croatia. *Long time sitting* was significant for both Romania (OR = 1.96, p = 0.01) and Turkey (OR = 0.49, p = 0.02), with opposite directions, indicating that Romanians are almost twice as likely to report sitting as a pain-increasing factor, while individuals in Turkey are about half as likely to do so. *Long time walking* was significantly more likely to be reported in Turkey (OR = 2.41, p < 0.01) but not significant in Romania.

In terms of models with weaker fit but statistical significance, *long time lying in a bed* had a moderate effect in Turkey (OR = 0.36, p = 0.01), showing that individuals from Turkey were less likely to report it as a pain-increasing factor compared to Croatia. Finally, no one from Romania marked *pushing an object* as a pain increasing factor and it approached significance for Turkey (p = 0.07). Table 8 provides the logistic regression model coefficients for country differences in pain self-coping and pain self-management factors. Again, age and BMI are not presented as they are used only as a controlling covariate. Among the pain self-coping factors, *doing exercise* was significantly less likely to be reported in Turkey compared to Croatia, with a large odds ratio (OR = 0.02, p < 0.01), indicating individuals in Turkey are far less likely to engage in this activity, with no one in Romania reporting *doing exercise* as a self-coping mechanism. *Taking a warm shower* was significantly more common in Romania (OR = 9.55, p < 0.01), with a very large effect size, but no significant difference was observed between Turkey and Croatia. *Heat application* was also more likely in both Romania (OR = 10.68, p < 0.01) and Turkey (OR = 3.87, p = 0.01), with Romania showing a stronger association. *Taking painkillers* was much less common in both Romania (OR = 0.03, p < 0.01) and Turkey (OR = 0.04, p < 0.01) compared to Croatia, indicating it is significantly underreported in these countries. For pain self-management, *physiotherapy* was more common in Romania (OR = 5.47, p < 0.01) and less common in Turkey (OR = 0.49, p = 0.01). *Osteopathy/manual therapy* and *spa and balneotherapy* were significantly more likely to be reported in Romania, with large ORs of 9.32 and 11.49, respectively (p < 0.01), while Turkey showed no significant differences for these factors. Finally, *complementary therapy* had an extraordinarily large OR of 56.74 in Romania (p < 0.01), though no significant difference was observed in Turkey.

**Table 8:** Logistic Regression Model Coefficients of Country Predictor for Pain Self-Coping & Pain Self-Management Factors

	Estimate	SE	Z	p	OR	LI	UI
**Pain self-Coping**							
**Doing exercise**							
Romania – Croatia	-	-	-	-	-	-	-
Turkey – Croatia	−4.02	1.03	−3.92	<0.01	0.02	0.00	0.13
**Taking a warm shower**							
Romania – Croatia	2.26	0.33	6.79	<0.01	9.55	4.98	18.33
Turkey – Croatia	−0.26	0.43	−0.62	0.54	0.77	0.33	1.77
**Taking a short walking around**							
Romania – Croatia	−1.05	0.32	−3.32	<0.01	0.35	0.19	0.65
Turkey – Croatia	−0.49	0.31	−1.59	0.11	0.61	0.34	1.12
**Heat application**							
Romania – Croatia	2.37	0.44	5.38	<0.01	10.68	4.51	25.30
Turkey – Croatia	1.35	0.48	2.82	0.01	3.87	1.51	9.91
**Cold application**							
Romania – Croatia	0.57	0.38	1.51	0.13	1.78	0.84	3.74
Turkey – Croatia	1.65	0.37	4.51	<0.01	5.19	2.54	10.62
**Taking painkillers**							
Romania – Croatia	−3.69	0.50	−7.39	<0.01	0.03	0.01	0.07
Turkey – Croatia	−3.33	0.44	−7.60	<0.01	0.04	0.02	0.08
**Pain self-Management**							
**Pharmacotherapy**							
Romania – Croatia	−0.49	0.29	−1.70	0.09	0.61	0.35	1.08
Turkey – Croatia	−0.70	0.30	−2.31	0.02	0.50	0.27	0.90
**Physiotherapy**							
Romania – Croatia	1.70	0.32	5.30	<0.01	5.47	2.92	10.26
Turkey – Croatia	−0.72	0.29	−2.47	0.01	0.49	0.28	0.86
**Osteopathy / manual therapy**							
Romania – Croatia	2.23	0.58	3.87	<0.01	9.32	3.01	28.84
Turkey – Croatia	0.66	0.66	1.01	0.31	1.94	0.53	7.09
**Spa and balneotherapy**							
Romania – Croatia	2.44	0.52	4.66	<0.01	11.49	4.12	32.07
Turkey – Croatia	0.58	0.61	0.95	0.34	1.78	0.54	5.83
**Complementary therapy**							
Romania – Croatia	4.04	1.04	3.88	<0.01	56.74	7.39	435.44
Turkey – Croatia	1.61	1.14	1.41	0.16	4.99	0.53	46.67

**Abbreviations**: Estimate, log odds ratio; SE, standard error (log odds ratio); Z, Z value; p, p-value; OR, odd ratio; LI, lower interval of 95% confidence interval (odds ratio); UI, upper interval of 95% confidence interval (odds ratio).

### Inferential Statistics: CDC Health Related Quality of Life-4

Table 9 shows the ordinal logistic regression model, examining the predictors of self-rated health status with variable CDC 1 (General Health Status) ordering of excellent, very good, good, fair, and poor.

**Table 9:** Descriptive Parameters and Testing of CDC Health-Related Quality of Life-4 by Country

	Croatia (N=131)	Romania (N=106)	Turkey (N=100)	Test p-value
**CDC 1**				OrdReg <0.01
Excellent	3 (2.3%)	0 (0%)	1 (1.0%)	
Very good	4 (3.1%)	9 (8.5%)	4 (4.0%)	
Good	63 (48.1%)	64 (60.4%)	29 (29.0%)	
Fair	38 (29.0%)	14 (13.2%)	59 (59.0%)	
Poor	23 (17.6%)	19 (17.9%)	7 (7.0%)	
**CDC 2**				KW <0.01
Mean (SD)	11.4 (11.1)	17.2 (9.72)	12.4 (9.38)	
Median [Min, Max]	5.00 [0, 30.0]	20.0 [0, 30.0]	10.0 [0, 30.0]	
**CDC 3**				KW <0.01
Mean (SD)	6.56 (9.26)	3.92 (5.96)	8.57 (9.66)	
Median [Min, Max]	3.00 [0, 30.0]	1.00 [0, 30.0]	5.00 [0, 30.0]	
**CDC 4**				KW <0.01
Mean (SD)	7.10 (10.3)	8.49 (9.41)	6.53 (8.39)	
Median [Min, Max]	2.00 [0, 30.0]	5.00 [0, 30.0]	3.00 [0, 30.0]	

**Abbreviations**: Min, Minimum; Max, Maximum; SD, Standard Deviation; OrdReg, ordinal regression (overall test for model: CDC 1 ~ Age + BMI + VAS + Country); KW, Kruskal–Wallis test.

The overall ordinal regression model fit was significant (χ² = 36.7, p < 0.01) with a McFadden R² of 0.045, indicating a low explanatory power. Significant predictors included VAS (χ² = 22.8, p < 0.01), with higher VAS scores being associated with worse self-rated health (OR = 1.30). Country also showed significance (χ² = 6.65, p = 0.04), with Romanian participants less likely to report better health compared to Croatians (OR = 0.57).

Kruskal–Wallis test has shown statistically significant differences for CDC 2, CDC 3, and CDC 4 between countries.

Table 10 has Dwass-Steel-Critchlow-Fligner post hoc pairwise comparisons. Pairwise comparisons using Dwass-Steel-Critchlow-Fligner tests show that Romania had significantly more days of poor physical health compared to both Croatia (p < 0.01) and Turkey (p < 0.01), while Turkey had significantly more days of poor mental health than Croatia (p = 0.03) and Romania (p < 0.01). For days of activity limitation, Romania had significantly more days than Croatia (p < 0.01).

**Table 10:** Dwass-Steel-Critchlow-Fligner Post Hoc Pairwise Comparisons

Country 1 vs Country 2		CDC 2 (Days)		CDC 3 (Days)		CDC 4 (Days)	
		W	p-value	W	p-value	W	p-value
Croatia	Romania	6.76	< 0.01	−1.16	0.69	4.87	< 0.01
Croatia	Turkey	2.42	0.2	3.64	0.03	1.25	0.65
Romania	Turkey	−5.23	< 0.01	5.6	< 0.01	−3.3	0.05

## Discussion

This study aimed to identify and describe the characteristics of chronic musculoskeletal pain, PM and coping strategies among OAs from Turkey, Croatia, and Romania. The study also aimed to explore cultural differences in pain experience and management, with particular emphasis on the influence of cultural, demographic, and health-related factors. In addition, we tried to show the cultural differences in terms of this point. This study highlights significant differences in demographic, socioeconomic, and HRQoL characteristics among OAs living in Croatia, Romania, or Turkey. For example, pain stigma affects coping strategies, with older adults using more passive coping methods as pain intensity increases,^52^ traditional and complementary medicine is common in Romania,^53^ and Croatia emphasizes biomedical treatments and physical activity.^54^ These findings provide valuable insights into how culture influences factors affecting pain such as increasing and decreasing factors in ageing, coping strategies with pain in ageing, PM strategies, and HRQoL.

Croatian participants were older on average compared to Romanian and Turkish participants, reflecting potentially greater experience with age-related conditions. Turkish participants, on the other hand, had the highest BMI, which may contribute to increased pain perception and physical dysfunction. Thus, this study provides an insight into the very different prevalence of obesity between countries, which may lead researchers to find the underlying reasons and consequently possible solutions with a comparison between countries.^55^,^56^

Education levels varied significantly across the countries, with Turkey having the lowest educational attainment. This, combined with a high proportion of housewives, suggests potential limitations in access to health information and structured PM strategies. The living arrangements also showed significant cultural influences, with most Turks living in their own residences compared to a smaller proportion in Croatians and Romanians, which could affect their access to social support systems.

CMP is a major health concern for OAs globally, with estimates suggesting that between 50% and 80% of elderly individual’s experience CMP.^55^ Research also shows that up to 75% of OAs suffer from CMP.^43^ Common causes of CMP include conditions such as arthritis and musculoskeletal disorders.^37^,^57^ In the study by Yagci et al, CMP was reported by 212 OAs (82,1%): 61,3% Spinal pain and 38,7% lower extremity pain.^57^ In some studies, OAs with CMP reported moderate pain (VAS score 5 to 6).^37^,^57^ Similarly, in our study, participants reported moderate pain. Pain triggers such as climbing stairs and prolonged sitting revealed important regional differences. Both Romanians and Turks reported climbing stairs as a significant pain-increasing factor. Interestingly, prolonged sitting was associated with increased pain among Romanians but was less likely to be reported in Turks, possibly due to differences in physical habits. Although national data indicate high rates of physical inactivity among older adults in Romania and Turkey,^58^ our findings suggest more complex behavioral patterns. For example, pain triggered by prolonged sitting was more common among Romanians, while prolonged walking was a more frequent trigger among Turks. These differences may reflect distinct physical habits and functional limitations rather than a uniformly inactive lifestyle across groups.^58^ These results show that the majority of OAs are inactive. In Turkey, prolonged walking was more likely to be a pain trigger, which might be linked to higher BMI among participants. These findings underline the need for context-specific interventions addressing behavioral factors.

The study revealed striking differences in CS and PM approaches. Croatians more frequently reported exercising as a PM strategy, indicating a potential preference for active interventions. In contrast, exercise was less commonly reported in Turkey, highlighting a potential area for health education and promotion.

Romanians favored traditional methods such as warm showers and heat applications for PM, alongside more structured approaches like physiotherapy, osteopathy/manual therapy, and spa/balneotherapy (S/BT). The extraordinarily high odds for complementary therapy (CT) use in Romania suggest a cultural inclination toward alternative health practices. Conversely, Turks showed a lower prevalence of these interventions, possibly due to cultural preferences.

Based on the published literature, health tourism services provided by spa resorts in Romania primarily focus on medical treatments and convalescence, catering mainly to the needs of OAs.^59^ Therefore, Romanians may have preferred balneology/spa therapy or warm showers. Massage therapy was preferred at the same rate. Physiotherapy was the most preferred treatment option by Romanians (83%). Croatians (48%) and Turks (31%) preferred it less than Romanians. O/MT, CT, acupuncture, and dry needling were the least preferred treatment options. These results clearly show that culture influences treatment preferences in the case of CMP. Treatment preferences revealed significant cultural differences. While medication use was the most common option across all three countries, Croatians reported the highest reliance (73.3%), followed by Turks (61.0%) and Romanians (58.5%). Physiotherapy was notably preferred by 83% of Romanian participants—substantially higher than among Croatians (48%) and Turks (31%). Use of complementary therapies (CT), including spa/balneotherapy and heat applications, was significantly more frequent in Romania, potentially reflecting cultural acceptance and access. Conversely, Turks reported the lowest usage of structured or alternative pain management interventions. These differences were supported by regression results and highlight the importance of culturally adapted educational strategies for effective pain management.

CMP highly affects HRQoL of AOs. It often leads to emotional distress as individuals’ experience increased levels of anxiety, depression, and frustration due to persistent discomfort.^19^ This can exacerbate physical difficulties. As a result, people with CMP may withdraw from social activities, leading to social isolation and further exacerbating mental health problems.^60^ Together, these factors create a vicious cycle that makes it difficult for people to break free from the grip of CMP and its negative impact on HRQoL. In our study, CDC HRQoL-4 results showed that Turks experienced more days of poor mental health. Romanians reported more days of poor physical health and activity limitations, which may suggest a higher self-perceived physical health burden; however, these findings should be interpreted with caution, as objective clinical data on comorbidities were not collected. Research on older adults in Turkey reveals significant mental health challenges. A study found 50% of low-income rural older adults exhibited high depressive symptoms, with women, widowed individuals, and those lacking health insurance at greater risk.^61^ Factors negatively affecting life satisfaction among Turkish older adults include poverty, poor health status, physical decline, and depression, while being married, higher education, and income-generating work positively impact satisfaction.^62^

These findings underscore the importance of culturally tailored interventions and healthcare policies. Educational campaigns promoting healthy lifestyle choices could benefit Turks, particularly targeting BMI reduction and encouraging exercise as a PM strategy. In Romania, the integration of CT into mainstream healthcare systems could improve accessibility and provide standardized care for individuals relying on these methods.

The experience of pain is characterized by enormous inter-individual variability driven by multiple bio-psycho-social factors. Individual differences in pain include the role of demographic, genetic, and psychosocial factors and their interactions. Cultural differences play a crucial role in the experiences and perceptions of CMP among OAs. Research showed that pain expression, interpretation, and management can differ widely across cultures, with varying cultural beliefs influencing how pain is perceived.^63^ These can impact treatment outcomes, as healthcare systems may adopt diverse approaches based on cultural norms. For instance, some cultures may emphasize stoicism and discourage the verbalization of pain, leading to underreporting, while others may focus more on holistic or alternative treatments for managing pain.^64^,^65^ Understanding these cultural nuances is essential for healthcare providers to deliver effective CMP strategies tailored to the needs of OAs from different cultural backgrounds.^66^ These support the idea that CMP perception is influenced by culture. Our study also yielded results that support this idea. Our findings not only aligned with the study’s objectives but also underscore important implications for healthcare policy and clinical practice. The observed cultural variations in pain perception, coping, and management strategies among older adults reflect broader societal influences, including differences in healthcare infrastructure, public health education, and economic accessibility. For example, the high reliance on complementary therapies in Romania highlights the potential for integrating such practices into formal healthcare systems to enhance accessibility and legitimacy. Policymakers should consider culturally tailored interventions that respect traditional practices while ensuring evidence-based care. Integrating multidisciplinary and culturally responsive pain management programs, including physiotherapy and lifestyle modification, and validated complementary approaches may significantly improve health-related quality of life for older adults, particularly in resource-limited settings.

Cultural variations in pain perception and coping are influenced by beliefs, traditions, and social norms.^67^,^68^ Kleinman’s explanatory models provide a framework for understanding these cultural differences, emphasizing the importance of culturally shaped beliefs in interpreting and expressing illness, including chronic pain.^69^ Cross-cultural studies reveal significant variations in pain perception, expression, and management across different cultural groups.^70^ Research indicates that cultural factors significantly influence mental health help-seeking behaviors, pain experiences, and healthcare disparities across countries. Hofstede’s cultural dimensions, particularly collectivism and uncertainty avoidance, shape how individuals perceive and express pain and mental health concerns.^71^,^72^ Cultural and ethnic backgrounds strongly affect pain perception, manifestation, and management, leading to disparities in acute pain treatment.^73^ The cultural construction of aging often normalizes pain, while active-aging ideologies may pressure older adults to conceal pain and ill-health.^11^ These findings underscore the need for culturally sensitive approaches in mental health technology design and pain management interventions to address health inequalities and improve patient outcomes across diverse populations.^71^,^73^

In their article, Moldovan et al present a practical, structured, and validated approach to addressing patient-centered issues in healthcare settings. This approach supports the goals of sustainability and social responsibility.^74^ It can be used to evaluate sustainable health services in various fields. It will be very useful to be used in studies similar to our study and to determine appropriate strategies.

The most powerful aspect of this study is that it shows the pain perception and PM preferences of OAs living in different countries regarding the common CMP problem of OAs. However, this study has some limitations: (1) this study used a convenience sampling method rather than random sampling. This approach may have introduced selection bias, as the sample may not be representative of the broader population of OAs with CMP. This limitation restricts the generalizability of the findings; (2) similarly, the second is that it involved 337 participants living in urban areas. Gender distribution was also unequal. Again, this further restricts the generalizability of the findings, as they may not fully reflect the PM strategies used by OAs in rural areas; (3) although HRQoL was assessed using four questions, a more specific HRQoL questionnaire related to pain and musculoskeletal disorders, such as SF-36, EuroQoL 5-Dimensions, WHOQOL-BREF, The Brief Pain Inventory or Musculoskeletal Health Questionnaire could provide deeper insights into the factors contributing to a lower HRQoL; (4) additionally, gathering data on the reasons for taking or not taking pain medication could offer a more comprehensive understanding of participants’ preferences; (5) Several logistic regression models, particularly those predicting pain-decreasing factors, exhibited low to moderate McFadden’s R² values, suggesting limited explanatory power and indicating that other relevant predictors may not have been captured; (6) The assumptions underlying the ordinal logistic regression model were formally tested, and the proportional odds assumption was found to be violated for the key categorical predictor “country.” While the overall model remains valid and interpretable, this violation should be interpreted with caution; (7) Due to concerns about sample size and stratification, gender was not included as a predictor in regression models. Although this decision was methodologically justified given our primary interest in cross-cultural patterns, it limits the study’s ability to examine gender-specific differences, which should be addressed in future research; (8) the questionnaire used in this study was developed for the specific context and population. While based on existing literature and clinical practice, it has not undergone formal psychometric validation; (9) finally, no data was collected on actual diagnoses or comorbid diseases, including cognitive decline. Participants stated only what they thought was their underlying issue for pain, which likely varies from what the actual cause is. Despite the limitations, the data could serve as a platform for improving existing prevention programs for CMP.

## Conclusions

The coping strategies and health-related quality of life of older adults are shaped not only by physical factors but also by beliefs and health perceptions that are unique to each country and influenced by culture. Education tailored to consider cultural context, including information on physical activity, medication use, and non-pharmacological methods such as physiotherapy, can enhance pain control and health among older adults.

Practitioners should be aware of the benefits of developing PM programs that consider cultural differences and the needs of each population. For example, encouraging exercise as a PM strategy through targeted educational campaigns could promote healthier lifestyles, reduce BMI, and enhance physical function. Similarly, integrating therapies such as balneotherapy into healthcare systems could improve accessibility for those who rely on these methods. Furthermore, efforts to educate OAs about safe and effective PM strategies, such as the benefits of physical activity, the appropriate use of pain medications, physiotherapy, and manual therapies, could enhance the effectiveness of coping with pain. As this study demonstrates, the results will help improve CMP management strategies for older adults by showing the effectiveness of different lifestyles and cultures in pain management.
